# Dementia And Physical Activity (DAPA) trial of moderate to high intensity exercise training for people with dementia: randomised controlled trial

**DOI:** 10.1136/bmj.k1675

**Published:** 2018-05-16

**Authors:** Sarah E Lamb, Bart Sheehan, Nicky Atherton, Vivien Nichols, Helen Collins, Dipesh Mistry, Sukhdeep Dosanjh, Anne Marie Slowther, Iftekhar Khan, Stavros Petrou, Ranjit Lall

**Affiliations:** 1 Centre for Rehabilitation Research and Centre for Statistics in Medicine, Nuffield Department of Orthopaedics Rheumatology & Musculoskeletal Sciences, Botnar Research Centre, University of Oxford, Oxford OX3 7LD, UK; 2 Warwick Clinical Trials Unit, University of Warwick, Coventry, UK; 3 Coventry and Warwickshire Partnership Trust, Coventry, UK; 4 Oxford University Hospitals NHS Foundation Trust, John Radcliffe Hospital, Oxford, UK; 5 Division of Health Sciences, University of Warwick, Coventry, UK

## Abstract

**Objective:**

To estimate the effect of a moderate to high intensity aerobic and strength exercise training programme on cognitive impairment and other outcomes in people with mild to moderate dementia.

**Design:**

Multicentre, pragmatic, investigator masked, randomised controlled trial.

**Setting:**

National Health Service primary care, community and memory services, dementia research registers, and voluntary sector providers in 15 English regions.

**Participants:**

494 people with dementia: 329 were assigned to an aerobic and strength exercise programme and 165 were assigned to usual care. Random allocation was 2:1 in favour of the exercise arm.

**Interventions:**

Usual care plus four months of supervised exercise and support for ongoing physical activity, or usual care only. Interventions were delivered in community gym facilities and NHS premises.

**Main outcome measures:**

The primary outcome was score on the Alzheimer’s disease assessment scale-cognitive subscale (ADAS-cog) at 12 months. Secondary outcomes included activities of daily living, neuropsychiatric symptoms, health related quality of life, and carer quality of life and burden. Physical fitness (including the six minute walk test) was measured in the exercise arm during the intervention.

**Results:**

The average age of participants was 77 (SD 7.9) years and 301/494 (61%) were men. By 12 months the mean ADAS-cog score had increased to 25.2 (SD 12.3) in the exercise arm and 23.8 (SD 10.4) in the usual care arm (adjusted between group difference −1.4, 95% confidence interval −2.6 to −0.2, P=0.03). This indicates greater cognitive impairment in the exercise group, although the average difference is small and clinical relevance uncertain. No differences were found in secondary outcomes or preplanned subgroup analyses by dementia type (Alzheimer’s disease or other), severity of cognitive impairment, sex, and mobility. Compliance with exercise was good. Over 65% of participants (214/329) attended more than three quarters of scheduled sessions. Six minute walking distance improved over six weeks (mean change 18.1 m, 95% confidence interval 11.6 m to 24.6 m).

**Conclusion:**

A moderate to high intensity aerobic and strength exercise training programme does not slow cognitive impairment in people with mild to moderate dementia. The exercise training programme improved physical fitness, but there were no noticeable improvements in other clinical outcomes.

**Trial registration:**

Current Controlled Trials ISRCTN10416500.

## Introduction

Nearly 47.5 million people worldwide have dementia.^1^ The challenge to families and health and social services is substantial.^1^ The hypothesis that aerobic and strengthening exercise might slow cognitive impairment in dementia has gained widespread popularity. Many studies describe plausible mechanisms using mammalian models, but there are fewer studies using human participants.^2^ ^3^

The results of recent systematic reviews of trials of exercise training in people with dementia have conflicted. One review concluded that exercise can improve physical but not cognitive impairment, neuropsychiatric symptoms, or health related quality of life.^4^ Another suggested that aerobic exercise has a positive effect on cognitive impairment, regardless of the type of dementia or dose of intervention.^5^ All reviews confirm the multiplicity of small studies of low methodological quality, limited duration of follow-up, and high unexplained heterogeneity in findings. In 2012, the UK government launched a prime minister’s challenge in which research to seek a cure for, or alleviation of, dementia symptoms was set as a national priority. The National Institute for Health Research (NIHR) commissioned the Dementia And Physical Activity (DAPA) trial to inform the debate about the potential benefit of exercise on cognitive impairment in people with dementia.

We compared the effect on cognitive impairment at 12 months of a combination of a moderate to high intensity aerobic and strength exercise training programme in addition to usual care compared with usual care alone in people with mild to moderate dementia. We designed and tested an intervention that targeted known mechanistic pathways in vascular and Alzheimer’s type dementia and which, if found effective, could be scaled for use within the UK National Health Service.

## Methods

### Study design

A full protocol has been published previously.^6^ This was a multicentre, pragmatic, investigator masked, randomised controlled trial. Random allocation was 2:1 in favour of the exercise arm. Participants were recruited from memory services in university and district general hospitals, NIHR dementia research registers and networks, and from primary care practices and community dementia services in 15 regions across England. Interventions were delivered in community gym facilities and occasionally in NHS facilities.

We recruited people with mild to moderate dementia and, when available, their primary carer. We asked carers to provide data about their relative or close friend with dementia for measures that specified the primary respondent should be the carer. For people with dementia, we determined capacity to consent in accordance with the principles of the Mental Capacity Act (2005) using research nurses and physiotherapists who received specific training. The assessment of capacity was based on a subjective opinion developed during the preliminary phases of enrolment. The nurses or physiotherapists made an assessment, using gentle questioning, to determine how much information was understood and processed. When people with dementia were assessed as having capacity, we obtained their informed consent. If people with dementia were assessed as lacking capacity, we asked the primary carer or personal consultee about the participant’s past and present wishes and feelings about taking part in research studies and we asked the primary carer or personal consultee to provide consent. If people with dementia were unable to give informed consent, and there was no carer or personal consultee, we sought nominated consultees who were well placed and prepared to act on behalf of potential participants (for example, a health professional independent of the study). We excluded people who lacked capacity and had no personal or nominated consultee. Carers gave their written informed consent to provide data about the person with dementia. We checked agreement for continued participation at each visit.

As a separate analysis, we asked carers to provide data on their own quality of life and caring experience. Carers provided separate written informed consent for the carer element of the study.

### Participants

People with dementia were eligible if they had a clinically confirmed diagnosis of dementia in accordance with the *Diagnostic and Statistical Manual of Mental Disorders*, fourth edition (DSM-IV)^7^ and a standardised mini mental state examination score (sMMSE)^8^ of greater than 10, were able to sit on a chair and walk 10 feet (3.05 m) without assistance, and lived in the community either alone or with others. We excluded people with acute, unstable physical or terminal illness that would make participation in the exercise programme unsafe.

### Study treatments

The interventions and rationale are described in detail elsewhere.^9^ Physiotherapists and exercise assistants prescribed and delivered interventions in the exercise arm. People with dementia attended an individual assessment where the prescription of aerobic and strength exercises was tailored to their fitness and health status. The assessment included a review of health conditions that required modifications of the exercise prescription (eg, diabetes, cardiovascular conditions, musculoskeletal conditions), and drugs that might be needed during sessions (eg. glyceryl trinitrate sprays, inhalers). Thereafter, people with dementia attended group sessions in a gym twice a week for four months; each session lasted 60 to 90 minutes. We also asked the participants to do home exercises for one additional hour each week during this period. The supervised programme lasted four months, after which we prescribed a more frequent home based programme with a target of unsupervised physical activity or exercise of 150 minutes each week (total). We used behavioural strategies (described elsewhere^9^) to promote adherence throughout, and up to three telephone motivational interviews were administered after the supervised programme. The behavioural strategies included guiding participants to choose home exercises or activities that matched their preferences for venue, personal situation, and ease of completion.

During the supervised period, people with dementia were overseen in groups of six to eight participants to minimise costs. In each group session, aerobic exercise consisted of static cycling with a five minute warm-up period followed by up to 25 minutes of moderate to hard intensity cycling, depending on tolerance level. We set target intensity using a six minute walk test according to Luxton^10^ and progressed the aerobic challenge using recognised methods.^9^ Strength training consisted of arm exercises using hand held dumb bells, including at least a biceps curl and, for more able individuals, shoulder forward raise, lateral raise, or press exercises, and leg strength training exercises using a sit-to-stand weighted vest (All Pro Exercise Products, FL) or a waist belt (Rehabus, Lerum, Sweden), or both. The starting weight for sit-to-stand varied between 0 and 12 kg depending on ability. The baseline target for strength training exercises was three sets of 20 repetitions. The sets had to be at least moderately difficult or hard to complete, and the weight was increased accordingly. In the ensuing sessions we added weight to ensure progression, with moderation of repetitions if needed. One physiotherapist and one assistant ran each session unless participation was low (≤3 participants) in which case one physiotherapist ran the session. In most instances, we provided consistency in staffing. Physiotherapists and assistants attended a one day training session that included techniques on communicating with and motivating people with dementia, and we provided a study manual. To ensure compliance with the treatment protocol we made regular visits for quality assurance.

All participants received usual care in accordance with clinical guidance that included counselling for carers and families, a clinical assessment, prescription of symptomatic treatments, and brief advice about physical activity.^11^ The participants’ doctors determined additional treatment on the basis of clinical need.

### Data collection

At the time of enrolment, trained interviewers who were registered health professionals checked eligibility and recorded demographic characteristics and the sMMSE score. Participants identified their gender as either female or male, and their ethnicity. We obtained baseline scores for outcome questionnaires before randomisation. We confirmed dementia diagnosis and extracted ICD (international classification of diseases, 10th revision, version 5) dementia diagnostic subcategory from hospital or primary care medical records.^12^ We followed-up all participants at six and 12 months after randomisation. Interviewers had regular quality assurance checks, with a member of the training team experienced with the ADAS-cog observing home interviews.

### Outcomes for people with dementia

Unless indicated the respondent is the person with dementia. The primary outcome was the Alzheimer disease assessment scale cognitive subscale^13^ (ADAS-cog 11 item scale, scored 0 to 70; higher scores indicate worse cognitive impairment) at 12 months. Secondary outcomes at six and 12 months after randomisation were measured using the Bristol activity of daily living index^14^ (scored 0 to 60, higher scores indicate worse impairment, carer rated), neuropsychiatric index^15^ (scored 0 to 144, higher scores indicate worse behavioural symptoms, carer rated), the three level version of the EQ-5D quality of life measure^16^ (scored 0 to 1, higher scores indicate better quality of life), the quality of life Alzheimer’s disease scale^17^ (scored 13 to 52, higher scores indicate better quality of life), ADAS-cog subscale at six months, and ADAS praxis, memory, and language subscales^13^ at six and 12 months (praxis scored 0-10, memory scored 0-35, language scored 0-25, higher scores indicate worse impairment). Carers provided a retrospective assessment of the participant’s falls and fractures for each six month follow-up period. We recorded deaths and data on use of healthcare and social care resources (including the number of physiotherapy sessions, exercise classes, or other structured physical activity programmes outside of the trial) using the client services receipt inventory^18^ (participant and carer rated). Follow-up interviews took about one and a half hours.

For each participant in the intervention arm we recorded the number of trial exercise sessions attended, the number of minutes spent cycling at low (warm-up), moderate, and high intensity, and the weight lifted and number of repetitions of each strength exercise. Participants repeated the six minute walk test six weeks after starting the classes and we calculated change in walking distance. We recorded the number of telephone contacts made after the end of the sessions and the adherence with physical activity recommendations reported during the course of the call.

At each session, physiotherapists asked participants and their carers if the person with dementia had experienced any adverse events since the last session. They also observed events during sessions, and during phone contacts asked about adverse events. Short episodes of muscular or postural soreness were expected. We defined serious adverse events as those that resulted in death, persistent or important disability or incapacity, were immediately life threatening, or required hospital admission or medical intervention to prevent one of the serious adverse events. We considered any event occurring during supervised or non-supervised exercise sessions and up to two hours after as being related to intervention.

### Carer outcomes

At each time point we measured carer burden with the Zarit burden interview^19^ (scored 0 to 88, higher scores indicate greater stress) and carer health related quality of life using the EQ-5D-3L.

### Randomisation and masking

An independent telephone randomisation system assigned participants to exercise training or usual care in a 2:1 ratio. Because 6-8 people with dementia had to be enrolled in each exercise session, we used unbalanced randomisation to minimise delays to starting exercise training. We provided the dates and times of sessions before enrolment, and we only randomised people who were able to attend these dates. If they could not attend these dates, then we delayed their enrolment, baseline assessment, and randomisation until the next cycle of exercise interventions became available. We stratified randomisation by region and used a minimisation algorithm within each region to balance the severity of dementia (either sMMSE 10-19 for moderate; ≥20 for mild)^8^ across the trial arms. An independent statistician used a computerised random number generator for the allocation sequence, then a central telephone registration and randomisation service implemented the sequence.

Masking participants, carers, or the teams providing intervention was not possible. Researchers unaware of treatment assignment undertook all baseline and follow-up interviews in the participants’ home. Before each interview, we asked participants and carers not to reveal the treatment they had received. If the allocation was revealed, we assigned a different interviewer to complete the next follow-up. Researchers who undertook data entry and cleaning were unaware of treatment allocation.

### Sample size

For the sample size calculation, we considered a between group difference of 2.45 ADAS-cog points in favour of exercise and a baseline standard deviation of 7.8 as the between group difference to be consistent with the conservative effects achieved by commonly used symptomatic drugs (giving a base target of 322).^20^ We inflated for unbalanced randomisation (n=38) and potential group (cluster) effects within the intervention arm (n=14, inflation factor 1.04), giving a target of 375 (α 0.05, power 80%). We assumed a 20% loss to follow-up (including 10% death), giving a minimum target of 468 (312 intervention: 156 control). P values are two sided. Because of the need to fill exercise cohorts we anticipated that the final sample would be greater than 468.

### Statistical analyses

We conducted analyses using intention to treat principles and analysed all people according to their random allocation. A few people with dementia withdrew themselves from the trial or were lost to follow-up. No people with dementia were withdrawn from the trial by investigators. We generated descriptive statistics for the randomised sample and the sample providing data for the primary analysis. Our prespecified strategy was to avoid imputation of missing cases if there were no discernible patterns in the missing data and the randomised and analysed samples were similar. During peer review a statistical reviewer asked for imputation of missing cases, and this is reported.

We used multilevel regression models with random effect for region to estimate treatment effects at each time point, negative binomial regression models for falls, and median regression for non-normally distributed data. We estimated clustering effects associated with exercise group membership. As cluster effects were negligible, we adjusted all estimates for baseline covariates (age, sex, cognitive impairment (sMMSE), region, and the variable being tested) only. Prespecified treatment subgroup analyses included pre-randomisation cognitive impairment (sMMSE ≥20 and <20), type of dementia (ICD clinical coding Alzheimer’s disease versus other), physical performance (EQ-5D mobility no problems versus some problems or confined to bed), and gender (male versus female). We analysed subgroups using statistical tests of interaction, including adjustment for baseline covariates.^21^ Compliance was defined a priori as attending 75% or more of group sessions (22 out of a maximum 30), consistent with most exercise guidelines.^9^ To estimate the effect of compliance on the primary outcome we used complier average causal effect (CACE) analysis.^22^ We calculated dose of aerobic and strength training for compliers, non-compliers, and overall. To test for differences in the baseline characteristics of compliers and non-compliers we used independent sample *t* tests or non-parametric equivalent.

We summarised the dose of aerobic training as the mean and standard deviation (or median and interquartile range for non-normal data) of the time spent in moderate and high intensity training. Also, as a proportion of the target time set by the physiotherapist before each session. For strength training the dose indicator was the difference between the average of the weight lifted for each exercise over the first four sessions (as it could take a few sessions to establish good form for maximum repetition) and the weight lifted during the final session. As we allowed for a decrease in repetitions to accommodate a greater strength load, we multiplied the amount of weight lifted by the number of repetitions to gain the overall weight lifted.

When available, we used the published recommendations for dealing with missing items within scales. We anticipated non-ignorable item missing data for the ADAS-cog^23^ and used recognised item level multiple imputation techniques, including baseline cognitive impairment, for the primary analysis.^24^ We undertook sensitivity analyses for missing ADAS-cog items, including worst score assignment and complete cases.^24^ In sensitivity analysis we used item response theory analysis^23^ but could not achieve an adequate fit to the data and therefore is not reported. We ran additional sensitivity analyses to inform whether unmasking of treatment assignment influenced the treatment estimate.

### Study monitoring

A trial steering and data monitoring committee reviewed safety, quality, and masked data at six monthly intervals, and approved changes to the statistical analysis plan and protocol. No interim analyses were done, but the trial steering committee/data monitoring committee could halt the trial for safety or ethical concerns. At the point of trial registration, the primary outcome was the mini mental state examination score. Before starting the trial we changed the primary outcome tool to the ADAS-cog. The reason was superior sensitivity to change that enabled a reduction in the sample size and greater comparability with other trials. We obtained appropriate permissions and paid any required fees for use of copyright protected materials.

### Patient involvement

The NIHR involved people with dementia and their representatives in specifying the question, including methods, selecting important outcome domains, and type of intervention through the commissioning process. The study team involved people with dementia, their representatives, and other stakeholders in the development of the intervention and protocol, including detailed feedback on the intervention, questionnaires, approach and invitation, acceptability of procedures, and logistics. Carers of people with dementia were formal members of the study trial steering committee/data monitoring committee. At the end of the study, people with dementia and carers were invited to a joint feedback day with research and clinical staff, and they contributed actively to discussions about the results and interpretation.

## Results

### Trial progression and recruitment

**Fig 1:**
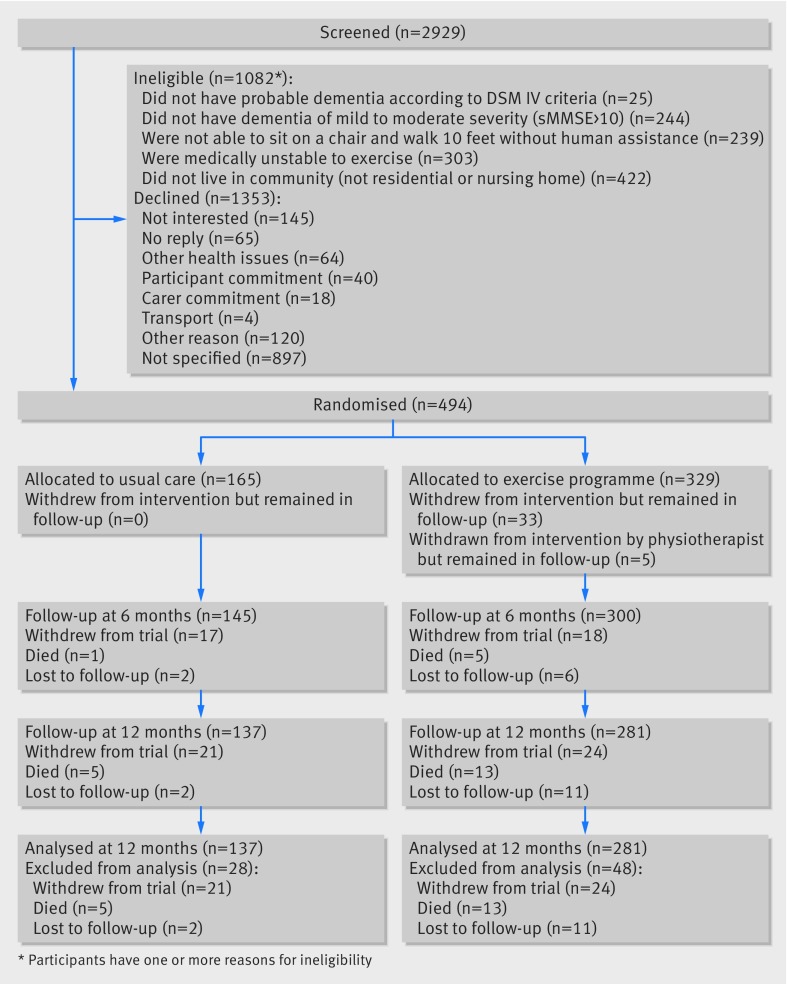
Flowchart of participants through trial. Participants could be ineligible for more than one reason

Between 1 February 2013 and 24 June 2015, 2929 people were screened, 1847 were found potentially eligible and approached, and 494 were randomised: 329 to an exercise programme and 165 to usual care (fig 1). A mean of 2 (SD 1.4) items from the Alzheimer’s disease assessment scale-cognitive subscale (ADAS-cog) were imputed for 18/137 (13%) of participants in the usual care arm and 33/281 (12%) in the exercise arm. Overall, deaths occurred in 5/165 (3%) participants in the usual care arm and 13/329 (4%) in the exercise arm. We carried out the exercise assessment at a median of 15 (interquartile range 9-25) days after randomisation, and the first session commenced at a median of 22 (14-33) days. The median time between attendance at the last exercise session and the first follow-up was 64 (50-80) days. Final follow-up was a median of 371 (365-379) days from randomisation, with no difference between groups.

### Sample

Overall, primary outcome data were available for 137/165 (83%) participants in the usual care arm and 281/329 (85%) in the exercise arm. Baseline characteristics for the randomised and analysed samples were well matched (table 1). Recruited men and women did not differ in age (mean difference −0.3 years, 95% confidence interval −1.69 to 1.16) or baseline ADAS-cog scores (−0.2, 95% confidence interval −1.86 to 1.41). More women than men lived alone (73/193 (38%) *v* 24/301 (8%), respectively). Most of the people with dementia were able to provide informed consent (376/484, 76%), some required a personal consultee (117/494, 24%), and one required a nominated consultee (1/494, 0.2%).

**Table 1:** Baseline demographic and clinical characteristics of randomised participants and those providing data for analysis of the primary end point (ADAS-cog score at 12 months). Values are numbers (percentages) unless stated otherwise

Characteristics	Randomised sample			Sample for primary analysis	
	Usual care (n=165)	Exercise programme (n=329)		Usual care (n=137)	Exercise programme (n=278)
Mean (SD) age (years)	78.4 (7.6)	76.9 (7.9)		78.1 (7.7)	76.9 (7.7)
Men	106 (64)	195 (59)		86 (63)	166 (60)
Living arrangements:					
Live alone	35 (21)	62 (19)		29 (21)	46 (16)
Live with relative, partner, or friends	130 (79)	267 (81)		108 (79)	232 (83)
Ethnicity:					
White	157 (95)	321 (98)		130 (95)	274 (99)
Other	8 (5)	8 (2)		7 (5)	4 (1)
Mean (SD) total No of drugs	5.5 (3.1)	5.7 (3.7)		5.6 (3.2)	5.5 (3.5)
Dementia drugs:					
Donepezil	84/155 (54)	166/318 (52)		70/129 (54)	148/270 (55)
Rivastigmine	0	6/318 (2)		0	3/270 (1)
Galantamine	1/155 (1)	6/318 (2)		0	0
Memantine	8/155 (5)	10/318 (3)		8/129 (6)	4/270 (1)
Mean (SD) ADAS-cog score	21.8 (7.7)	21.4 (9.6)		21.4 (7.8)	21.2 (9.5)
Median (interquartile range) language subscale score	2 (1-4)	2 (0-4)		2 (1-4)	2 (0-4)
Mean (SD) memory subscale score	17.4 (4.8)	16.7 (6.2)		17.1 (4.9)	16.6 (6.1)
Median (interquartile range) praxis subscale score	1 (1-2)	1 (1-2)		1 (1-2)	1 (1-2)
Mean (SD) sMMSE score	21.6 (4.6)	22.0 (4.7)		22.1 (4.6)	22.1 (4.6)
Mean (SD) EQ-5D (self report) score	0.85 (0.18)	0.82 (0.20)		0.86 (0.16)	0.84 (0.19)
Mean (SD) QoL-AD (self report) score	39.3 (5.2)	38.7 (5.6)		39.4 (5.0)	39.1 (5.4)
Median (interquartile range) NPI (proxy report) score	10 (3-20)	7.5 (3.0-17.5)		10 (3-19)	8 (3-17)
Median (interquartile range) BADL (proxy report) score	10 (5-16)	11 (6-17)		9.5 (5-15)	10.5 (5-17)
Fallen in past 6 months	56/154 (36)	90/305 (30)		41/129 (32)	70/258 (27)
Mean (SD) No of falls in past 6 months	2.8 (4.9)	2.7 (3.3)		3.1 (5.5)	2.8 (3.7)
Broken bones in past 6 months	5/154 (3)	9/305 (3)		2/129 (2)	9/258 (3)
Mean (SD) carer age (years)	70.2 (10.5)	69.1 (11.4)		70.1 (10.4)	69.8 (10.7)
Male carer	29/154 (19)	87/305 (28.5)		25/129 (19)	74/258 (29)
Carer relationship:					
Spouse	117/154 (76)	239/305 (78)		98/129 (76)	209/258 (81)
Son or daughter (in law)	32/154 (21)	55/305 (18)		27/129 (21)	42/258 (16)
Other	4/154 (3)	11/305 (4)		3/129 (2)	7/258 (3)
Frequency of caring:					
4-7 days a week	127/154 (82)	268/305 (88)		105/129 (81)	226/258 (88)
Less than once a month	9/154 (6)	8/305 (3)		7/129 (5)	7/258 (3)
Mean (SD) ZBI score	29.0 (15.7)	30.6 (15.4)		28.5 (15.7)	30.2 (15.0)
Mean (SD) carer EQ-5D score	0.82 (0.23)	0.79 (0.21)		0.81 (0.23)	0.79 (0.21)

ADAS-cog=Alzheimer’s disease assessment scale-cognitive subscale; sMMSE=standardised mini mental state examination; EQ-5D=European quality of life measure; Qol-AD=quality of life Alzheimer’s disease scale; NPI=neuropsychiatric index; BADL=Bristol activity of daily living index; ZBI=Zarit burden interview.

### Intervention

Table 2 shows data on the profile of participants in the exercise arm, attendance, dose of exercise delivered, and physical fitness outcomes by compliance status. Overall, 21 physiotherapists and 17 assistants delivered 1697 face to face training sessions. Most of the participants assigned to the exercise arm (317/329, 96%) attended the physiotherapy assessment, and the proportion assessed as having comorbidity was high (278/317, 88%). The median group size was 6 (interquartile range 5-7). More than 65% (214/329) of the participants were classed as compliers. Men were more likely to comply than women, otherwise there were no differences. Weight lifted improved in all strengthening exercises across the sessions, as did the duration of higher intensity aerobic activity. Over six weeks, the distance walked in six minutes improved by 18.1 m (95% confidence interval 11.6 m to 24.6 m; P<0.001). Most of the participants (245/329, 75%) received three motivational telephone calls after the sessions finished and 217/245 (88%) reported continuing with exercise at home.

**Table 2:** Intervention data by compliance status. Values are numbers (percentages) unless stated otherwise

Participants	Compliers (n=214)	Non-compliers (n=115)	All (n=329)
**Demographics**			
Mean (SD) age (years)	76.4 (7.8)	77.7 (8.1)	76.9 (7.9)
Men	144 (67)	51 (44)	195 (59)
Mean (SD) ADAS-cog score (imputed)	21.9 (9.7)	20.5 (9.4)	21.4 (9.6)
Medical conditions*			
Heart or circulatory	102 (48)	52 (50)	154 (49)
Glyceryl trinitrate spray	18 (8)	13 (13)	31 (10)
Lung disease	22 (10)	18 (17)	40 (13)
Inhaler	22 (10)	14 (14)	36 (11)
Diabetes	38 (18)	21 (20)	59 (19)
Neurological condition	42 (20)	15 (15)	57 (18)
Limiting joint or muscle pain	117 (55)	60 (58)	177 (56)
Broken bones in past 6 months	14 (6.5)	8 (8)	22 (7)
Mental illness	65 (30)	45 (44)	110 (35)
**Dose received†**			
Mean (SD) sessions attended (range)	26.2 (2.1) (22-30)	11.2 (8.0) (0-22)	21.0 (8.7) (0-30)
Sit to stand:			
Median (interquartile range) start weight (kg)	4 (1.7-6)	3 (1.4-6)	4 (1.5-6)
Median (interquartile range) finish weight (kg)	7 (0-15.1)	6 (1-10)	6.8 (0-12.1)
Median difference (95% CI): difference between start and finish weight (kg)	4 (2.6 to 5.6)	2.1 (0.9 to 3.2)	3 (1.8 to 4.2)
Median (interquartile range) total weight lifted (kg×reps)‡	3460.8 (1857.3-5537.1)	1307.5 (276-2284)	2569.8 (1231.4-4672)
Median (interquartile range) arm exercises (total weight lifted)‡	2469.4 (1626.3-3444.2)	1001.9 (463-1574)	1933.5 (1105-2905)
Cycling:			
Mean (SD) total mins low intensity	210.5 (70.7)	131.5 (72.6)	186.6 (79.9)
Median (interquartile range) total mins high intensity	58 (19-98)	0 (0-18)	38 (2-78)
Moderate or high exercise (total) in last session (mins):			
Median (interquartile range) target No	20 (10-20)	15 (5-20)	20 (10-20)
Median (interquartile range) actual No	20 (10-20)	10 (0-20)	17 (7-20)
**6 minute walk test**			
Mean (SD) baseline 6 min walk distance (m)	340.0 (114.0)	315.4 (108.7)	332.1 (112.7)
Mean (SD) 6 week 6 min walk distance (m)	363.0 (118.1)	355.8 (101.6)	361.8 (115.3)
Mean difference 0-6 weeks (95% CI) in 6 min walk distance (m)	19.6 (12.5 to 26.7)	10.7 (−6.3 to 27.8)	18.1 (11.6 to 24.6)

For numbers and corresponding percentages, denominator is total in column heading unless stated otherwise.

* Of 329 participants randomised to exercise programme arm, 317 attended pre-exercise assessment.

† Of 329 participants randomised to exercise programme arm, 306 had resistance session data.

‡ Total weight lifted is sum of (weight lifted multiplied by number of repetitions) across all sessions.

### Usual care and other interventions

No important differences were found in health and social care resource use (see appendix 1). Less than 1% (3/415) of the participants used structured exercise outside of the trial prescription, less than 4% (15/415) reported attending a physiotherapist’s clinic, and nobody randomised to usual care accessed trial exercise sessions.

### Adverse events

Twenty five adverse events occurred (eight were possibly related, nine probably related, and eight definitely related) and four serious related adverse events (one hospital admission for exercise induced angina, two injurious falls, and one case of substantially worsening hip pain) in the exercise arm and no reports in the usual care arm. In the exercise arm an adverse event was reported by 23/329 (7.0%, 95% confidence interval 4.7% to 10.3%) participants.

### Outcomes

**Fig 2:**
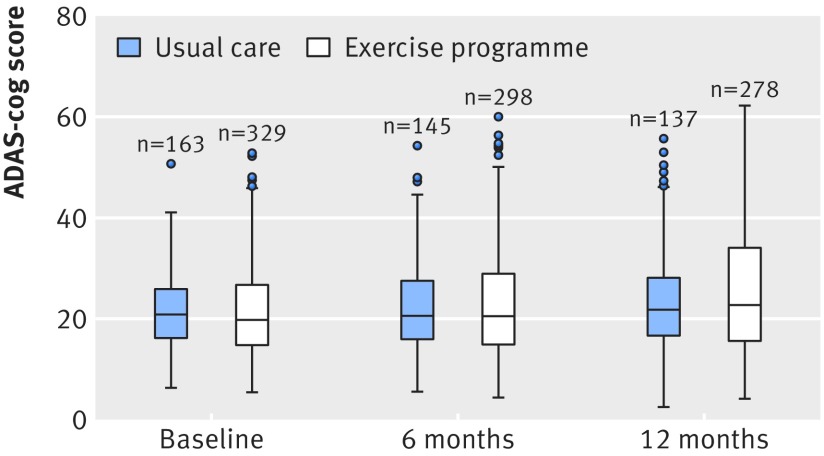
Box plots of raw data for Alzheimer’s disease assessment scale-cognitive subscale (ADAS-cog) at baseline and six and 12 months. Data are median (central line), interquartile range (box margins), adjacent values (whiskers), and outliers (dots)

Table 3 and figure 2 show the treatment effect estimates. Cognitive impairment declined over the 12 month follow-up in both trial arms. The exercise arm had higher global ADAS-cog scores at 12 months (adjusted mean difference −1.4, 95% confidence interval −2.6 to −0.2). Higher scores indicate worse cognition, although the average difference was less than the prespecified between group difference, and clinical relevance was uncertain. Sensitivity analyses for the primary outcome were consistent in the direction of effect regardless of the method of accounting for missing data at item level (table 3). Imputation for missing cases yielded a complete intention to treat estimate of adjusted mean difference −1.3 (95% confidence interval −2.4 to −0.2). No evidence was found of differences in other secondary outcomes, including the rate of falls over 12 months (incident rate ratio 1.1, 95% confidence interval 0.8 to 1.6; P=0.69).

**Table 3:** Main estimates of treatment effect

Outcomes	6 months						12 months				
	Usual care		Exercise programme		Adjusted estimate (95% CI); P value		Usual care		Exercise programme		Adjusted estimate (95% CI); P value
	No	Mean (SD)	No	Mean (SD)			No	Mean (SD)	No	Mean (SD)	
ADAS-cog: cognitive subscale	145	22.4 (9.4)	298	22.9 (11.6)	−0.6 (−1.6 to 0.4); 0.24		137	23.8 (10.4)	278	25.2 (12.3)	−1.4 (−2.6 to −0.2); 0.03
Sensitivity analyses:											
Complete case analysis	135	21.4 (8.5)	280	21.7 (10.3)	−0.7 (−1.7 to 0.4); 0.20		119	22.4 (9.7)	245	22.9 (10.6)	−1.7 (−3.0 to −0.4); 0.01
Worst score analysis	145	23.8 (12.8)	298	23.3 (12.5)	0.4 (−0.8 to 1.7); 0.52		137	25.5 (13.7)	278	26.6 (14.8)	−0.9 (−2.6 to 0.7); 0.27
ADAS-cog subscales:											
Language*	145	2 (1 to 5)	299	2 (0 to 5)	0.001 (−0.4 to 0.54); 1.00		137	2 (0.7 to 5)	280	2 (1 to 7)	−0.2 (−0.86 to 0.45); 0.61
Memory	145	17.3 (5.6)	298	17.3 (6.9)	−0.5 (−1.2 to 0.3); 0.22		137	18.1 (5.6)	279	18.5 (6.7)	−0.8 (−1.6 to 0.02); 0.06
Praxis*	145	2 (1 to 3)	299	1 (1 to 3)	−0.001 (−0.2 to 0.2); 1.00		137	1 (1 to 3)	281	2 (1 to 3.9)	0.1 (−0.4 to 0.2); 0.38
EQ-5D:											
Score (self report)	139	0.83 (0.21)	292	0.80 (0.21)	0.02 (−0.01 to 0.06); 0.24		131	0.82 (0.25)	261	0.81 (0.22)	−0.002 (−0.04 to 0.04); 0.93
VAS score (self report)	138	78.7 (18.8)	288	75.4 (20.6)	−0.1 (−3.6 to 3.4); 0.94		124	78.3 (19.4)	261	75.5 (19.3)	1.4 (−2.4 to 5.2); 0.46
Score (proxy report)	134	0.65 (0.29)	277	0.64 (0.27)	−0.01 (−0.06 to 0.03); 0.53		128	0.60 (0.32)	259	0.60 (0.28)	−0.02 (−0.07 to 0.03); 0.43
EQ-5D VAS (proxy report)	135	65.4 (20.5)	278	66.1 (20.1)	−0.6 (−4.3 to 3.1); 0.74		128	65.6 (19.9)	260	65.0 (20.0)	1.2 (−2.4 to 4.8); 0.52
EQ-5D score (carer report)	132	0.77 (0.24)	277	0.76 (0.23)	−0.004 (−0.04 to 0.03); 0.84		129	0.78 (0.23)	261	0.76 (0.24)	−0.002 (−0.04 to 0.04); 0.94
EQ-5D VAS (carer report)	136	72.4 (20.7)	277	73.4 (19.7)	−1.4 (−4.7 to 1.8); 0.38		129	75.1 (18.7)	261	74.5 (18.6)	0.2 (−2.9 to 3.3); 0.90
ZBI (carer report)	122	32.9 (17.1)	273	33.9 (16.0)	0.06 (−2.0 to 2.1); 0.96		125	32.7 (16.6)	256	34.5 (16.1)	−0.5 (−2.8 to 1.7); 0.64
QoL-AD:											
Score (self report)	124	39.0 (5.9)	263	38.9 (6.1)	−0.1 (−1.0 to 0.8); 0.88		119	39.1 (5.7)	237	38.4 (5.8)	0.7 (−0.2 to 1.7); 0.13
Score (proxy report)	114	31.3 (6.2)	239	31.6 (6.2)	0.1 (−0.9 to 1.0); 0.89		118	30.6 (6.0)	234	30.6 (6.1)	0.02 (−1.0 to 1.0); 0.96
NPI (proxy report)*	110	8.5 (3 to 22)	234	12 (4 to 21)	−0.6 (−3.1 to 2.1); 056		105	9 (3 to 20) 13.5 (13.1)	215	12 (4 to 23)	−2.1 (−4.8 to 0.7); 0.14
BADL (proxy report)*	129	14.6 (10.4)	271	14.6 (9.5)	0.8 (−0.3 to 2.0); 0.15		124	15.9 (9.7)	251	17.0 (10.2)	0.3 (−1.7 to 1.2); 0.70

ADAS-cog=Alzheimer’s disease assessment scale-cognitive subscale; EQ-5D=European quality of life measure; VAS=visual analogue scale; ZBI=Zarit burden interview; Qol-AD=quality of life Alzheimer’s disease scale; NPI=neuropsychiatric index; BADL=Bristol activity of daily living index.

Estimates are random effect models adjusted for age, sex, site, baseline mini mental state examination score and baseline value of the outcome variable being estimated.

* Data are not normally distributed estimates and are obtained from median regression. Descriptive statistics are median and interquartile range.

The complier average causal effect estimate for the primary outcome was −2.0 (95% confidence interval −3.87 to −0.22) indicating worse cognitive impairment (below the prespecified between group difference) in those with higher attendance of sessions. No differences were found in carer burden or quality of life between the two interventions. There were no statistically significant subgroup effects (table 4).

**Table 4:** Subgroup analyses of 12 month ADAS-cog (imputed) outcome. Values are number of participants, mean (standard deviation) unless stated otherwise

Subgroups	Usual care			Exercise programme			Within stratums: effect estimate (95% CI)*	Interaction effect (95% CI)	P value for interaction
	Baseline	12 months		Baseline	12 months				
Sex:									
Male	85; 21.7 (8.4)	86; 23.9 (11.4)		168; 20.6 (8.7)	166; 23.9 (11.8)		−1.2 (−2.78 to 0.46)	−0.6 (−3.17 to 1.88)	0.62
Female	50; 20.7 (6.8)	51; 23.7 (8.5)		113; 22.2 (10.4)	112; 27.3 (12.9)		−1.8 (−3.60 to 0.08)		
sMMSE score:									
<20	36; 30.5 (7.6)	36; 34.3 (10.9)		85; 31.1 (8.3)	84; 37.7 (10.8)		−2.8 (−5.32 to −0.27)	1.8 (−0.98 to 4.50)	0.21
≥20	99; 18.0 (4.6)	101; 20.1 (7.2)		196; 17.0 (6.1)	194; 19.8 (8.4)		−0.9 (−2.32 to 0.46)		
EQ-5D-3L mobility score:									
No problems walking	102; 21.9 (8.1)	103; 24.5 (10.5)		207; 22.5 (9.6)	204; 26.6 (12.8)		−1.3 (−2.65 to 0.10)	0.00005 (−2.86 to 2.86)	1.00
Some problems/confined to bed	32; 19.5 (6.5)	33; 21.7 (10.0)		74; 18.0 (8.1)	74; 21.4 (10.0)		−1.6 (−4.21 to 1.00)		
Type of dementia:									
Alzheimer’s disease	107; 20.9 (7.9)	108; 23.7 (10.0)		229; 21.5 (9.5)	227; 25.6 (12.4)		−1.1 (−2.41 to 0.29)	1.3 (−1.81 to 4.35)	0.42
Other (mixed, vascular, other types)	28; 23.2 (7.5)	29; 24.5 (11.8)		52; 20.1 (8.9)	51; 23.5 (11.9)		−2.7 (−5.58 to 0.16)		

sMMSE=standardised mini mental state examination; EQ-5D-3L=three level Euorpean quality of life measure.

* Adjusted estimates from multilevel regression models with random effect for region and adjustment for age, sex, sMMSE score, and baseline value of dependent variable.

At 12 months, interviewers reported that they knew the treatment assignment in 11/132 cases (8%) in the usual care arm and 94/276 (33%) in the exercise arm (overall 26%). In the usual care arm 10 of the 11 (90%) assignments were identified correctly and in the exercise arm this was 90 out of 94 (96%) assignments. Including masking status in the covariate adjustment did not alter the treatment effect estimate.

## Discussion

A four month aerobic and strengthening exercise programme of moderate to high intensity added to usual care does not slow cognitive decline in people with mild to moderate dementia. The exercise improved physical fitness in the short term, but this did not translate into improvements in activities of daily living, behavioural outcomes, or health related quality of life. There is the possibility that the intervention could worsen cognition.

### Comparison with previous studies

The results disagree with several small studies.^4^ ^5^ Most previous studies are single centre and many have uncertain allocation concealment and poor masking of outcome assessment.^4^ ^5^ Smaller studies are more likely to draw erroneous conclusions. Previous studies might not have achieved as high a dose of exercise and some have mixed exercise and cognitive training making it difficult to isolate the effectiveness of different elements of the training programme. For example, the Finnish Alzheimer disease exercise (FINALEX) study compared a 12 month combined executive, strength and balance exercise programme at either home or in a group supervised setting versus usual care in people with mild to moderate dementia.^25^ Combined home exercise and cognitive training had a benefit on functional independence, a marginal benefit on executive function and falls, but no benefits on global cognitive function or mobility.^25^ Several other relatively large studies reported null effects on global cognition.^4^ ^5^

### Strengths and limitations of this study

In comparison with previous trials,^4^ ^5^ we recruited a substantially larger sample size, used a measure of cognitive impairment recommended as a core outcome in consensus guidelines,^26^ ^27^ and maintained high levels of follow-up. We used robust allocation concealment and masked outcome assessment. Loss to follow-up was low and baseline characteristics for the randomised and analysed samples were similar. We conducted a range of sensitivity analyses. Analysis of both observed and fully imputed data on cognitive impairment yielded a similar treatment effect estimate. No suggestion of a hidden effect in subgroups of mobility, cognition, sex, and underlying cause of dementia was found. As expected, the 95% confidence intervals within each subgroup were broader but consistent with the overall finding. We delivered a relatively high dose of exercise, with good compliance. The dose of strengthening exercise was at least equal to previous work in older people without dementia,^28^ and much higher than that achieved in residential care settings where people are more frail and a similar intervention was found to improve balance but not cognitive or activities of daily living status.^29^

Participants and carers were not masked to allocation, but this is an unavoidable limitation. We were successful in masking three quarters of interviews for the primary time point. The level of unmasking was higher in the exercise arm but it seems unlikely that this would account for our findings. Physical fitness improved,^30^ but we are limited to data in the exercise arm and during the structured exercise intervention period only. Hence we cannot conclude definitively that the intervention improves physical fitness. It is unlikely that fitness would have improved in the usual care arm as there was no evidence of engagement in exercise or physiotherapy. Subgroup analyses might be underpowered as the proportions in the various stratums were not balanced.^21^ We asked carers to recall falls events instead of using monthly calendar diaries. This might result in underestimation of the overall number of falls but is unlikely to affect the between group difference. We collected data on adverse events related to exercise in the intervention arm only. The period of structured exercise in our study might have been too short to produce positive benefits. We believe this unlikely as changes in physical fitness had occurred during the intervention but did not transfer into other clinically meaningful benefits. Withdrawal of the structured exercise programme by six months might have led to an accelerated decline in cognitive impairment mediated through social and affective mechanisms.

The numbers of people who declined participation in the trial was high and suggests that exercise might not be an attractive proposition. We recruited more men than women even though dementia is more common in women in western Europe.^1^ Compliance was better in men. Women might have found the offer or experience of exercise unattractive. Alternatively, more women were living alone, with the associated difficulties of motivating and getting themselves to sessions. We did not include an attention control, as our intention was for a pragmatic trial.

### Interpretation

The study was designed as a superiority trial. Slowing cognitive decline was an ambitious target, but we anticipated at least a benefit in functioning in activities of daily living given broader knowledge about the effects of improved physical fitness.^28^ It is possible that cognition might have been worsened by the intervention. Whether the effect on cognitive impairment we observed is important is uncertain. We did not prespecify a value for a negative effect, but the average effect observed was smaller than our prespecified superiority target of 2.45 ADAS-cog points. An influential international consensus group suggests that a between group difference of 2 points (or 25%) might be worthwhile depending on the cost and safety profile of the intervention.^27^ Treatments in common use are associated with differences smaller than 2 points.^20^ In the context of the overall average annual decline in cognitive impairment, the decline in the usual care arm was consistent with published expectations,^31^ and the treatment difference was at least half as much as the annual rate of decline. Survival is on average 4.5 years in mild to moderate dementia.^31^ Worsening in cognitive impairment was greater in those who complied with the intervention and is physiologically feasible. High intensity training in healthy humans can have negative short term effects, including slow reoxygenation of cortical areas with a transient reduction in executive function.^32^ Inflammation induced by higher levels of exercise might also be implicated.^33^

### Recommendations and policy implications

Moderate to high intensity aerobic and strength exercise cannot be recommended as a treatment option for cognitive impairment in dementia. Future trials should explore other forms of exercise, including psychomotor protocols that are commonly used in long term neurological conditions where the primary intent is improving physical functioning. Investigators should consider the possibility that some types of exercise intervention might worsen cognitive impairment and of a “rebound” effect if the exposure to an intervention is to be time limited.

This exercise programme is not an effective way to manage cognitive impairment, functional impairment, or behavioural disturbances in older people with mild to moderate dementia.

### Conclusion

A four month period of moderate to high intensity aerobic and strength exercise training, and ongoing support to exercise does not slow cognitive decline and might worsen cognitive impairment in people with mild to moderate dementia. Although moderate to high intensity exercise improves physical fitness, no clinical outcomes that we studied responded in a positive direction.

**What is already known on this topic**

The role of exercise in slowing cognitive decline in people with dementia is uncertain
There is a paucity of randomised controlled trials of sufficient size and methodological quality to inform practice

**What this study adds**

People with mild to moderate dementia can engage and comply with moderate to high intensity aerobic and strengthening exercise and improve physical fitness
These benefits do not, however, translate into improvements in cognitive impairment, activities in daily living, behaviour, or health related quality of life
The exercise programme might possibly have worsened cognitive impairment

## Web extra

Extra material supplied by authors

## Notes

Contributors: SEL (chief investigator): conceived and designed the study and had overall responsibility for the study design and delivery. She is the guarantor. DM did the statistical analysis. NA developed and implemented the intervention and was training and quality assurance lead for the intervention. SD was responsible for the trial logistics and management, and writing and reviewing of the manuscript. IK was responsible for the economic analysis of the trial. RL is a grant holder and was responsible for the statistical analysis. HC was responsible for recruitment and guidance on strategies for recruitment and testing procedures. AMS is a grant holder and lead ethicist and provided the framework for consent and advice and guidance on ethical issues during the trial. VN was responsible for trial management and was the recruitment lead and was responsible for training and quality assurance. SP is a grant holder and was responsible for the economic analysis and writing and reviewing of the manuscript. BS was responsible for study design and conduct, clinical responsibility, and writing and reviewing of the manuscript. All authors have contributed to drafting the manuscript and approving the final version. The corresponding author attests that all listed authors meet authorship criteria and that no others meeting the criteria have been omitted.

Ethical approval: This study was approved by national ethics review committees (REC No 11/SW/0232) and research governance departments in each organisation.

Data sharing: The relevant anonymised patient level data, full dataset, technical appendix, and statistical code are available on reasonable request from the corresponding author. Consent for data sharing was not obtained but the presented data are anonymised and risk of identification is low.

Transparency: The lead author (SEL) affirms that the manuscript is an honest, accurate, and transparent account of the study being reported; that no important aspects of the study have been omitted; and that any discrepancies from the study as planned (and, if relevant, registered) have been explained.

## References

[1] Prince MJ World Alzheimer Report 2015: the global impact of dementia: an analysis of prevalence, incidence, cost and trends. Alzheimers Disease International. ADI, 2015.

[2] Gallaway PJ Miyake H Buchowski MS Physical Activity: A Viable Way to Reduce the Risks of Mild Cognitive Impairment, Alzheimer’s Disease, and Vascular Dementia in Older Adults. Brain Sci 2017;7:22. 10.3390/brainsci7020022 28230730 PMC5332965

[3] Moore KM Girens RE Larson SK A spectrum of exercise training reduces soluble Aβ in a dose-dependent manner in a mouse model of Alzheimer’s disease. Neurobiol Dis 2016;85:218-24. 10.1016/j.nbd.2015.11.004 26563933

[4] Forbes D Forbes SC Blake CM Thiessen EJ Forbes S Exercise programs for people with dementia. Cochrane Database Syst Rev 2015;(4):CD006489. 25874613 10.1002/14651858.CD006489.pub4 PMC9426996

[5] Groot C Hooghiemstra AM Raijmakers PG The effect of physical activity on cognitive function in patients with dementia: A meta-analysis of randomized control trials. Ageing Res Rev 2016;25:13-23. 10.1016/j.arr.2015.11.005 26607411

[6] Atherton N Bridle C Brown D Dementia and Physical Activity (DAPA) - an exercise intervention to improve cognition in people with mild to moderate dementia: study protocol for a randomized controlled trial. Trials 2016;17:165. 10.1186/s13063-016-1288-2 27015659 PMC4807539

[7] American Psychiatric Association Diagnostic and statistical manual of mental disorders (DSM-5®). American Psychiatric Publishing, 2013.

[8] Vertesi A Lever JA Molloy DW Standardized Mini-Mental State Examination. Use and interpretation. Can Fam Physician 2001;47:2018-23. 11723596 PMC2018449

[9] Brown D Spanjers K Atherton N Development of an exercise intervention to improve cognition in people with mild to moderate dementia: Dementia And Physical Activity (DAPA) Trial, registration ISRCTN32612072. Physiotherapy 2015;101:126-34. 10.1016/j.physio.2015.01.002 25724322

[10] Luxton N Alison JA Wu J Mackey MG Relationship between field walking tests and incremental cycle ergometry in COPD. Respirology 2008;13:856-62. 10.1111/j.1440-1843.2008.01355.x 18811884

[11] National Institute for Clinical Excellence (NICE). Donepezil, galantamine, rivastigmine and memantine for the treatment of Alzheimer’s disease. *NICE technology appraisal guidance* 2011;**217**.

[12] World Health Organization The ICD-10 classification of mental and behavioural disorders: clinical descriptions and diagnostic guidelines Version 5. World Health Organization, 2010.

[13] Mohs RC Knopman D Petersen RC Development of cognitive instruments for use in clinical trials of antidementia drugs: additions to the Alzheimer’s Disease Assessment Scale that broaden its scope. The Alzheimer’s Disease Cooperative Study. Alzheimer Dis Assoc Disord 1997;11(Suppl 2):S13-21. 10.1097/00002093-199700112-00003 9236948

[14] Bucks RS Ashworth DL Wilcock GK Siegfried K Assessment of activities of daily living in dementia: development of the Bristol Activities of Daily Living Scale. Age Ageing 1996;25:113-20. 10.1093/ageing/25.2.113 8670538

[15] Cummings JL Mega M Gray K Rosenberg-Thompson S Carusi DA Gornbein J The Neuropsychiatric Inventory: comprehensive assessment of psychopathology in dementia. Neurology 1994;44:2308-14. 10.1212/WNL.44.12.2308 7991117

[16] EuroQol Group EuroQol--a new facility for the measurement of health-related quality of life. Health Policy 1990;16:199-208. 10.1016/0168-8510(90)90421-9 10109801

[17] Logsdon RG Gibbons LE McCurry SM Teri L Quality of life in Alzheimer’s disease: patient and caregiver reports. J Ment Health Aging 1999;5:21-32.

[18] Beecham JK, Knapp M. Costing psychiatric interventions http://www.dirum org/instruments/details/44: DIRUM; 1992. Accessed 26 June 2015.

[19] Zarit SH Reever KE Bach-Peterson J Relatives of the impaired elderly: correlates of feelings of burden. Gerontologist 1980;20:649-55. 10.1093/geront/20.6.649 7203086

[20] Kaduszkiewicz H Zimmermann T Beck-Bornholdt HP van den Bussche H Cholinesterase inhibitors for patients with Alzheimer’s disease: systematic review of randomised clinical trials. BMJ 2005;331:321-7. 10.1136/bmj.331.7512.321 16081444 PMC1183129

[21] Brookes ST Whitley E Peters TJ Mulheran PA Egger M Davey Smith G Subgroup analyses in randomised controlled trials: quantifying the risks of false-positives and false-negatives. Health Technol Assess 2001;5:1-56. 10.3310/hta5330 11701102

[22] Dunn G Maracy M Dowrick C ODIN group Estimating psychological treatment effects from a randomised controlled trial with both non-compliance and loss to follow-up. Br J Psychiatry 2003;183:323-31. 10.1192/bjp.183.4.323 14519610

[23] Verma N Beretvas SN Pascual B Masdeu JC Markey MK Alzheimer’s Disease Neuroimaging Initiative New scoring methodology improves the sensitivity of the Alzheimer’s Disease Assessment Scale-Cognitive subscale (ADAS-Cog) in clinical trials. Alzheimers Res Ther 2015;7:64. 10.1186/s13195-015-0151-0 26560146 PMC4642693

[24] Little RJA Rubin DB Statistical Analysis with Missing Data. John Wiley & Sons, Inc, 1986.

[25] Bohannon RW Crouch R Minimal clinically important difference for change in 6-minute walk test distance of adults with pathology: a systematic review. J Eval Clin Pract 2017;23:377-81. 10.1111/jep.12629 27592691

[26] Webster L Groskreutz D Grinbergs-Saull A Core outcome measures for interventions to prevent or slow the progress of dementia for people living with mild to moderate dementia: Systematic review and consensus recommendations. PLoS One 2017;12:e0179521. 10.1371/journal.pone.0179521 28662127 PMC5491018

[27] Pitkälä KH Pöysti MM Laakkonen ML Effects of the Finnish Alzheimer disease exercise trial (FINALEX): a randomized controlled trial. JAMA Intern Med 2013;173:894-901. 10.1001/jamainternmed.2013.359 23589097

[28] Bean JF Herman S Kiely DK Increased Velocity Exercise Specific to Task (InVEST) training: a pilot study exploring effects on leg power, balance, and mobility in community-dwelling older women. J Am Geriatr Soc 2004;52:799-804. 10.1111/j.1532-5415.2004.52222.x 15086665

[29] Toots A Littbrand H Lindelöf N Effects of a High-Intensity Functional Exercise Program on Dependence in Activities of Daily Living and Balance in Older Adults with Dementia. J Am Geriatr Soc 2016;64:55-64. 10.1111/jgs.13880 26782852 PMC4722852

[30] Elder GA Gama Sosa MA De Gasperi R Transgenic mouse models of Alzheimer’s disease. Mt Sinai J Med 2010;77:69-81. 10.1002/msj.20159 20101721 PMC2925685

[31] Sudo M Komiyama T Aoyagi R Nagamatsu T Higaki Y Ando S Executive function after exhaustive exercise. Eur J Appl Physiol 2017;117:2029-38. 10.1007/s00421-017-3692-z 28780602

[32] Cipryan L Tschakert G Hofmann P Acute and Post-Exercise Physiological Responses to High-Intensity Interval Training in Endurance and Sprint Athletes. J Sports Sci Med 2017;16:219-29. 28630575 PMC5465984

[33] Valenzuela M Brayne C Sachdev P Wilcock G Matthews F Medical Research Council Cognitive Function and Ageing Study Cognitive lifestyle and long-term risk of dementia and survival after diagnosis in a multicenter population-based cohort. Am J Epidemiol 2011;173:1004-12. 10.1093/aje/kwq476 21378129

