# Supplementary material for: Dementia And Physical Activity (DAPA) trial of moderate to high intensity exercise training for people with dementia: randomised controlled trial
Source: BMJ. 2018 May 16;361:k1675. doi: 10.1136/bmj.k1675 (PMC5953238; doi:10.1136/bmj.k1675)
Supplement: Supplementary file 1 — Supplementary information: appendix 1 [file lams041769.ww1.pdf]

Appendix 1: Health Resource Use by Trial Allocation, Category and Study Period for Complete Cases

Data are the mean number of contacts for Baseline (three months prior to randomisation) and between randomisation and 6 months, 6 and 12 month follow up.

Data are the average number of contacts per person, and the number of people having that type of contact. The denominator is the number of complete CRFs at that time point.

Contacts with less than 1% of use have been excluded to ease reading unless they are about exercise or physiotherapy.

Three months prior to baseline

Resource Category (Unit)	Exercise<br>Mean (SE) n#(%)	Usual Care<br>Mean (SE) n#(%)
Baseline		
Patient Accommodation (# nights)	N=326	N=162
Acute psychiatric ward	0.0(0.03) 3(0.9%)	0.1(0.06) 3(1.9%)
Hospital Services	N=327	N=162
General medical ward (Days)	0.2(0.06) 12(3.7%)	0.1(0.06) 4(2.5%)
Acute psychiatric ward (Days)	0.1(0.03) 5(1.5%)	0.0(0.02) 3(1.9%)
Other hospital in- patient ward (Days)	0.6(0.06) 103(31.5%)	0.7(0.13) 54(33.3%)
Out- Patient services (Appointments)	0.0(0.01) 15(4.6%)	0.1(0.02) 8(4.9%)
Accident and Emergency (Appointments)	0.1(0.04) 9(2.8%)	0.0(0.01) 2(1.2%)
Other	0.0(0.02) 9(2.8%)	0.0(0.02) 5(3.1%)
Day Care Services	N=325	N=162
Local Authority Social Serv. (1/2 Days)	0.1(0.04) 6(1.8%)	0.1(0.05) 6(3.7%)
Voluntary/Private (1/2 Days)	0.1(0.04) 19(5.8%)	0.5(0.33) 8(4.9%)
NHS (not hospital) (1/2 Days)	0.1(0.04) 9(2.8%)	0.0(0.02) 2(1.2%)
Lunch Club (Visits)	0.1(0.02) 10(3.1%)	0.0(0.01) 1(0.6%)
Social Club (Visits)	0.0(0.01) 9(2.8%)	0.1(0.08) 6(3.7%)
Other	0.0(0.01) 12(3.7%)	0.2(0.15) 6(3.7%)
General Community Health Services (#visits)	N=326	N=162
Geriatrician – Office Visit	0.0(0.01) 5(1.5%)	0.0(0.01) 2(1.2%)
General practitioner – Office Visit	1.1(0.08) 187(57.4%)	1.2(0.13) 94(58.0%)

Resource Category (Unit)	Exercise<br>Mean (SE) n#(%)	Usual Care<br>Mean (SE) n#(%)
General practitioner – Home Visit	0.1(0.03) 8(2.5%)	0.0(0.02) 6(3.7%)
Practice nurse (GP clinic) – Office Visit	0.7(0.13) 91(27.9%)	0.6(0.10) 47(29.0%)
Practice nurse (GP clinic) – Home Visit	0.0(0.00) 2(0.6%)	0.0(0.01) 2(1.2%)
District nurse– Office Visit	0.0(0.02) 2(0.6%)	0.0(0.01) 2(1.2%)
District nurse– Home Visit	0.0(0.02) 6(1.8%)	0.0(0.01) 2(1.2%)
Incontinence nurse – Office Visit	0.0(0.01) 4(1.2%)	0.0(0.02) 2(1.2%)
Occupational therapist – Home Visit	0.0(0.02) 5(1.5%)	0.0(0.02) 3(1.9%)
Physiotherapist – Office Visit	0.1(0.03) 8(2.5%)	0.0(0.04) 2(1.2%)
Physiotherapist – Home Visit	0.0(0.01) 3(0.9%)	0.1(0.07) 3(1.9%)
Other- Office Visit	0.1(0.03) 20(6.1%)	0.1(0.05) 11(6.8%)
Other- Home Visit	0.0(0.01) 3(0.9%)	0.0(0.02) 3(1.9%)
Other:		
Exercise Class/Physical Activity	0.0(0.00) 2(0.6%)	0.0(0.04) 2(1.2%)
Optician	0.1(0.04) 9(2.8%)	0.1(0.07) 3(1.9%)
Dentist	0.1(0.02) 10(3.1%)	0.1(0.07) 3(1.9%)
Community Mental Health Services (#visits)	N=326	N=162
CPN/CMHN–Office Visit	0.1(0.02) 33(10.1%)	0.1(0.02) 11(6.8%)
CPN/CMHN– Home Visit	0.2(0.10) 34(10.4%)	0.1(0.07) 12(7.4%)
Community psychiatrist– Office Visit	0.3(0.03) 75(23.0%)	0.3(0.05) 41(25.3%)
Community psychiatrist– Home Visit	0.0(0.01) 9(2.8%)	0.0(0.02) 5(3.1%)
Psychologist – Office Visit	0.0(0.01) 7(2.1%)	0.0(0.00) 0(0.0%)
Psychologist – Home Visit	0.0(0.00) 2(0.6%)	0.0(0.02) 2(1.2%)
Other – Office Visit	0.1(0.02) 9(2.8%)	0.2(0.15) 2(1.2%)
Other – Home Visit	0.1(0.10) 5(1.5%)	0.0(0.04) 2(1.2%)
Social Care Services (# visits)	N=326	N=161
Social worker – Home	0.0(0.02) 9(2.8%)	0.0(0.01) 5(3.1%)
Home care worker – Home	0.7(0.41) 7(2.1%)	2.6(2.44) 2(1.2%)
Carer worker – Home	0.1(0.06) 5(1.5%)	0.0(0.01) 1(0.6%)

Resource Category (Unit)	Exercise<br>Mean (SE) n[#](%)	Usual Care<br>Mean (SE) n[#](%)
Chiropodist – Office	0.1(0.02) 17(5.2%)	0.0(0.02) 4(2.5%)
Chiropodist – Home	0.0(0.02) 6(1.8%)	0.1(0.03) 5(3.1%)
Self-help group– Office	0.1(0.05) 7(2.1%)	0.1(0.03) 5(3.1%)
Self-help group carer – Office	0.0(0.01) 3(0.9%)	0.1(0.05) 3(1.9%)
Other – Office	0.1(0.06) 8(2.5%)	0.1(0.05) 4(2.5%)
Other – Home	0.0(0.02) 5(1.5%)	0.0(0.02) 3(1.9%)
Equipment, Adaptations / Repairs (n %)	N=325	N=162
Health Service	0.03(0.01) 11 (3.4%)	0.06(0.02) 10 (6.2%)
Voluntary organization	0.0(0.00) 1 (0.3%)	0.01(0.01) 2 (1.2%)
Self financed	0.04(0.01) 14 (4.3%)	0.04(0.01) 6 (3.7%)
Private organization	0.05(0.01) 19 (5.8%)	0.11(0.02) 16 (9.9%)
Privately Provided General Community Health Services[a]	N=326	N=162
	0.1 (0.03) 10 (3.0%)	0.0(0.03) 2 (1.2%)

Baseline to 6 months

Resource Category (Unit)	Exercise<br>Mean (SE) n*(%)	Usual Care<br>Mean (SE) n*(%)
Baseline to 6 Months		
Patient Accommodation (# nights)	N=298	N=142
Acute psychiatric ward	0.2(0.08) 10(3.4%)	0.2(0.15) 2(1.4%)
Hospital Services	N=298	N=141
General medical ward (Days)	0.5(0.14) 22(7.4%)	0.3(0.16) 6(4.3%)
Acute psychiatric ward (Days)	0.3(0.14) 11(3.7%)	0.2(0.14) 6(4.3%)
Other hospital in- patient ward (Days)	1.2(0.18) 120(40.3%)	1.2(0.20) 59(41.8%)
Out- Patient services (Appointments)	0.1(0.02) 30(10.1%)	0.1(0.02) 10(7.1%)
Accident and Emergency (Appointments)	0.1(0.03) 19(6.4%)	0.1(0.03) 8(5.7%)
Day hospital (Days)	0.0(0.01) 6(2.0%)	0.0(0.01) 2(1.4%)
Other	0.2(0.06) 13(4.4%)	0.1(0.03) 6(4.3%)
Day Care Services	N=298	N=141
Local Authority Social Serv. (1/2 Days)	0.0(0.02) 6(2.0%)	0.2(0.07) 8(5.7%)
Voluntary/Private (1/2 Days)	0.2(0.04) 27(9.1%)	0.1(0.04) 6(4.3%)
Lunch Club (Visits)	0.0(0.02) 8(2.7%)	0.0(0.02) 5(3.5%)
Social Club (Visits)	0.0(0.01) 11(3.7%)	0.2(0.17) 5(3.5%)
Other	0.1(0.02) 10(3.4%)	0.1(0.06) 10(7.1%)
General Community Health Services (#visits)	N=297	N=141
General practitioner – Office Visit)	2.0(0.15) 205(69.0%)	1.9(0.24) 93(66.0%)
General practitioner – Home Visit	0.1(0.02) 16(5.4%)	0.0(0.02) 4(2.8%)
Practice nurse (GP clinic) – Office Visit	1.3(0.25) 130(43.8%)	1.2(0.27) 61(43.3%)
Practice nurse (GP clinic) – Home Visit	0.0(0.01) 3(1.0%)	0.0(0.04) 2(1.4%)
District nurse– Home Visit	0.2(0.17) 9(3.0%)	0.0(0.01) 2(1.4%)
Incontinence nurse – Office visit	0.0(0.01) 5(1.7%)	0.0(0.02) 2(1.4%)
Incontinence nurse – Home Visit	0.0(0.01) 3(1.0%)	0.0(0.01) 1(0.7%)
Occupational therapist – Home Visit	0.0(0.01) 6(2.0%)	0.1(0.04) 8(5.7%)

Resource Category (Unit)	Exercise<br>Mean (SE) n*(%)	Usual Care<br>Mean (SE) n*(%)
Physiotherapist – Office visit	0.1(0.06) 12(4.0%)	0.0(0.02) 4(2.8%)
Physiotherapist – Home Visit	0.1(0.04) 6(2.0%)	0.0(0.02) 2(1.4%)
Other - Office Visit	0.1(0.04) 21(7.1%)	0.3(0.12) 13(9.2%)
Other:		
Exercise Class/Physical Activity	0.0(0.01) 2(0.7%)	0.0(0.01) 1(0.7%)
Optician	0.2(0.17) 9(3.0%)	0.0(0.02) 3(2.1%)
Dentist	0.1(0.06) 12(4.0%)	0.0(0.02) 4(2.8%)
Community Mental Health Services (#visits)	N=298	N=141
CPN/CMHN–Office Visit	0.1(0.02) 29(9.7%)	0.1(0.03) 15(10.6%)
CPN/CMHN– Home Visit	0.3(0.17) 30(10.1%)	0.3(0.11) 11(7.8%)
Community psychiatrist– Office Visit	0.2(0.03) 56(18.8%)	0.2(0.04) 29(20.6%)
Community psychiatrist– Home Visit	0.0(0.01) 6(2.0%)	0.0(0.01) 4(2.8%)
Psychologist – Office Visit	0.0(0.02) 3(1.0%)	0.2(0.11) 2(1.4%)
Psychologist – Home Visit	0.0(0.01) 2(0.7%)	0.1(0.06) 2(1.4%)
Other – Office Visit	0.1(0.05) 12(4.0%)	0.5(0.35) 5(3.5%)
Other – Home Visit	0.3(0.25) 9(3.0%)	0.0(0.03) 4(2.8%)
Social Care Services (# visits)	N=298	N=141
Care manager – Home	0.0(0.01) 3(1.0%)	0.0(0.01) 1(0.7%)
Social worker – Home	0.1(0.02) 16(5.4%)	0.1(0.05) 7(5.0%)
Home care worker – Home	1.9(1.32) 3(1.0%)	0.3(0.34) 1(0.7%)
Carer worker – Home	1.5(0.72) 11(3.7%)	0.7(0.74) 2(1.4%)
Chiropodist – Office	0.1(0.03) 13(4.4%)	0.2(0.07) 12(8.5%)
Chiropodist – Home	0.0(0.02) 7(2.3%)	0.0(0.03) 2(1.4%)
Other – Office	0.2(0.11) 6(2.0%)	0.0(0.02) 3(2.1%)
Other – Home	0.0(0.02) 4(1.3%)	0.0(0.01) 2(1.4%)
Equipment, Adaptations / Repairs (n %)	N=298	N=141
Health Service	0.04(0.01) 13 (4.4%)	0.06(0.02) 9 (6.3%)
Local Authority	0.01(0.01) 3 (1.0%)	0.01(0.01) 1 (0.7%)

Resource Category (Unit)	Exercise<br>Mean (SE) n*(%)	Usual Care<br>Mean (SE) n*(%)
Self financed	0.04(0.01) 13 (4.4%)	0.06(0.02) 9 (6.3%)
Private organization	0.13(0.02) 38 (12.8%)	0.13(0.03) 18 (12.7%)
Privately Provided General Community Health Services[a]	N=297	N=141
Physiotherapist – Office visit	0.0(0.01) 1 (0.3%)	0.0(0.01) 1 (0.7%)
Physiotherapist – Home Visit	0.0(0.00) 0 (0.0%)	0.0(0.00) 0 (0.0%)
Other – Office Visit	0.0(0.01) 4 (1.3%)	0.4(0.21) 5 (3.5%)

6 to 12 months

Resource Category (Unit)	Exercise<br>Mean (SE) n*(%)	Usual Care<br>Mean (SE) n*(%)
Patient Accommodation (# nights)	N=280	N=136
Care home providing personal care	0.5(0.24) 4(1.4%)	0.1(0.05) 2(1.5%)
Dual Registered home (providing both personal and nursing care)	0.2(0.13) 3(1.1%)	0.0(0.00) 0(0.0%)
Acute psychiatric ward	1.0(0.39) 15(5.4%)	0.9(0.70) 2(1.5%)
Hospital Services	N=280	N=135
General medical ward (Days)	1.1(0.40) 28(10.0%)	1.4(0.74) 13(9.6%)
Acute psychiatric ward (Days)	0.1(0.11) 3(1.1%)	0.0(0.00) 0(0.0%)
Other hospital in-patient ward (Days)	1.0(0.13) 102(36.4%)	1.1(0.18) 53(39.3%)
Outpatient services (Appointments)	0.1(0.03) 29(10.4%)	0.1(0.02) 9(6.7%)
Accident and Emergency (Appointments)	0.1(0.05) 13(4.6%)	0.1(0.02) 8(5.9%)
Day hospital (Days)	0.0(0.01) 3(1.1%)	0.0(0.01) 3(2.2%)
Other	0.3(0.17) 8(2.9%)	0.0(0.01) 2(1.5%)
Day Care Service	N=280	N=135
Local Authority Social Serv. (1/2 Days)	0.0(0.02) 8(2.9%)	0.1(0.05) 8(5.9%)
Voluntary/Private (1/2 Days)	0.2(0.04) 35(12.5%)	0.2(0.13) 10(7.4%)
NHS (not hospital) (1/2 Days)	0.0(0.00) 0(0.0%)	0.1(0.04) 3(2.2%)
Lunch Club (Visits)	0.1(0.02) 9(3.2%)	0.0(0.01) 4(3.0%)
Social Club (Visits)	0.1(0.06) 9(3.2%)	0.1(0.06) 3(2.2%)
Other	0.2(0.08) 24(8.6%)	0.1(0.06) 7(5.2%)
General Community Health Services (#visits)	N=280	N=135
General practitioner – Office Visit	1.8(0.14) 184(65.7%)	1.7(0.27) 86(63.7%)
General practitioner – Home Visit	0.1(0.03) 19(6.8%)	0.2(0.10) 13(9.6%)
Practice nurse (GP clinic) – Office Visit	1.0(0.24) 109(38.9%)	0.8(0.13) 53(39.3%)
Practice nurse (GP clinic) – Home Visit	0.1(0.05) 5(1.8%)	0.2(0.19) 2(1.5%)
District nurse– Office Visit	0.1(0.04) 5(1.8%)	0.0(0.00) 0(0.0%)

Resource Category (Unit)	Exercise<br>Mean (SE) n*(%)	Usual Care<br>Mean (SE) n*(%)
District nurse– Home Visit	0.3(0.18) 10(3.6%)	0.1(0.09) 2(1.5%)
Incontinence nurse – Office Visit	0.0(0.01) 7(2.5%)	0.0(0.01) 2(1.5%)
Incontinence nurse – Home Visit	0.0(0.01) 7(2.5%)	0.1(0.03) 5(3.7%)
Occupational therapist – Home Visit	0.1(0.02) 11(3.9%)	0.1(0.04) 2(1.5%)
Physiotherapist – Office visit	0.1(0.04) 8(2.9%)	0.2(0.09) 7(5.2%)
Physiotherapist – Home Visit	0.1(0.04) 7(2.5%)	0.1(0.06) 3(2.2%)
Other		
Exercise Class/Physical Activity	0.3(0.22) 2(0.7%)	0.0(0.01) 1(0.7%)
Optician	0.1 (0.05) 12 (4.3%)	0.0(0.01) 4(3.0%)
Dentist	0.2 (0.04) 14 (5.0%)	0.1(0.03) 5(3.7%)
Office Visit	0.2(0.04) 26(9.3%)	0.1(0.05) 6(4.4%)
Home Visit	0.5(0.40) 12(4.3%)	0.5(0.44) 2(1.5%)
Community Mental Health Services (#visits)	N=280	N=136
CPN/CMHN–Office Visit	0.1(0.02) 27(9.6%)	0.1(0.03) 18(13.3%)
CPN/CMHN– Home Visit	0.5(0.29) 29(10.4%)	0.1(0.06) 7(5.2%)
Community psychiatrist– Office Visit	0.1(0.02) 28(10.0%)	0.2(0.04) 19(14.1%)
Community psychiatrist– Home Visit	0.1(0.02) 9(3.2%)	0.0(0.01) 2(1.5%)
Psychologist – Office Visit	0.0(0.00) 0(0.0%)	0.1(0.10) 2(1.5%)
Other – Office Visit	0.1(0.06) 6(2.1%)	0.2(0.15) 2(1.5%)
Other – Home Visit	0.0(0.01) 3(1.1%)	0.1(0.11) 3(2.2%)
Social Care Services (# visits)	N=280	N=135
Care manager – Home	0.0(0.01) 4(1.4%)	0.0(0.01) 2(1.5%)
Social worker – Home	0.1(0.04) 19(6.8%)	0.2(0.05) 14(10.4%)
Home care worker – Home	3.0(1.69) 9(3.2%)	8.7(4.91) 7(5.2%)
Carer worker – Home	2.4(1.29) 8(2.9%)	1.3(1.24) 2(1.5%)
Chiropodist – Office	0.1(0.04) 14(5.0%)	0.2(0.06) 13(9.6%)
Chiropodist – Home	0.0(0.02) 2(0.7%)	0.3(0.22) 2(1.5%)
Sitting scheme – Home	0.1(0.11) 3(1.1%)	0.4(0.36) 1(0.7%)

Resource Category (Unit)	Exercise Mean (SE) n*(%)	Usual Care Mean (SE) n*(%)
Other – Office	0.1(0.04) 5(1.8%)	0.3(0.23) 2(1.5%)
Other – Home	0.8(0.67) 3(1.1%)	0.2(0.19) 2(1.5%)
Equipment, Adaptations / Repairs (n %)	N=280	N=136
Self	0.08(0.02) 21 (7.5%)	0.11(0.03) 15 (11.0%)
Health Service	0.08(0.02) 22 (7.9%)	0.22(0.04) 30 (22.1%)
Local Authority	0.02(0.01) 6 (2.1%)	0.07(0.02) 9 (6.6%)
Voluntary	0.0(0.00) 0 (0.00)	0.03(0.01) 4 (2.9%)
Private	0.17(0.02) 48 (17.1%)	0.2(0.03) 27 (19.9%)
Privately Provided General Community Health Services[a]	N=280	N=135
Other – Office Visit	0.1(0.09) 5(1.8%)	0.0(0.02) 3(2.2%)

[*] number of participants who used a health resource at least once at a given assessment

[a] consisting of private health professional visits (GP, alternative health and physiotherapist)
